# CIB1 and CIB2 are HIV-1 helper factors involved in viral entry

**DOI:** 10.1038/srep30927

**Published:** 2016-08-04

**Authors:** Ana Godinho-Santos, Allan J. Hance, João Gonçalves, Fabrizio Mammano

**Affiliations:** 1 Research Institute for Medicines (iMed.ULisboa), Faculty of Pharmacy, University of Lisbon, Lisbon, Portugal; 2 INSERM, U941, Paris, F-75010, France; 3 Univ Paris Diderot, Sorbonne Paris Cité, F-75475, Paris, France

## Abstract

HIV-1 relies on the host-cell machinery to accomplish its replication cycle, and characterization of these helper factors contributes to a better understanding of HIV-host interactions and can identify potential novel antiviral targets. Here we explored the contribution of CIB2, previously identified by RNAi screening as a potential helper factor, and its homolog, CIB1. Knockdown of either CIB1 or CIB2 strongly impaired viral replication in Jurkat cells and in primary CD4+ T-lymphocytes, identifying these proteins as non-redundant helper factors. Knockdown of CIB1 and CIB2 impaired envelope-mediated viral entry for both for X4- and R5-tropic HIV-1, and both cell-free and cell-associated entry pathways were affected. In contrast, the level of CIB1 and CIB2 expression did not influence cell viability, cell proliferation, receptor-independent viral binding to the cell surface, or later steps in the viral replication cycle. CIB1 and CIB2 knockdown was found to reduce the expression of surface molecules implicated in HIV-1 infection, including CXCR4, CCR5 and integrin α4β7, suggesting at least one mechanism through which these proteins promote viral infection. Thus, this study identifies CIB1 and CIB2 as host helper factors for HIV-1 replication that are required for optimal receptor-mediated viral entry.

Human Immunodeficiency Virus type-1 (HIV-1) depends on the host cell machinery to support its replication, and is able to exploit a variety of cellular factors and pathways. Understanding how cellular proteins promote HIV-1 infection provides both insights into the cellular mechanisms underlying individual steps of retroviral replication, and may permit the identification of new therapeutic targets^1^,^2^. One frequently used approach for identifying host proteins important in HIV-1 replication has been the use of small interfering RNA (siRNA) and short hairpin RNA (shRNA) screens^3^,^4^,^5^,^6^,^7^. A large number of candidate proteins have been identified in these studies. For example, in three of these screens^3^,^6^,^7^, >3% of all human protein-coding genes were identified at least once. The overlap between different studies, however, has generally been relatively low (<10%), and it has been suggested that it may be useful to focus attention of these “overlap” candidates, which may constitute a more extensively corroborated set of putative host factors assisting HIV replication^8^. In a recent iterative shRNA screen performed in one of our laboratories, the calcium- and integrin-binding 2 (CIB2) protein was identified^4^, a protein that had previously been detected in an independent screen^6^. Little is known about the expression and function of CIB2, but more information is available for CIB1, another family member that shares 60% homology with CIB2^9^. Thus, in the current study assessing the importance of proteins of this family in HIV-1 replication, we included both CIB1 and CIB2 in our evaluation.

CIB1 and CIB2 belong to a family of EF-hand proteins that consists of four members in humans (CIB1, -2, -3 and -4)^10^. As their name suggests, these proteins bind Ca2+ (and Mn2+), both of which can induce conformational changes, as well as the α-chain of integrin heterodimers. Early studies suggested that CIB1 and CIB2 might target only certain specific integrins^11^,^12^, but the strong conservation of the consensus CIB1 binding site in all α-integrin chains and the demonstration by immunoprecipitation and competitive binding assays of interactions with many integrins has led to the prediction that at least CIB1 could bind to all 24 known integrin heterodimers^13^. At the mRNA level, both CIB1 and CIB2 are widely expressed in human tissues^9^,^14^,^15^, although CIB1 expression is generally greater than that of CIB2. In contrast, the expression of CIB3 and CIB4 has a more restricted distribution, and only low levels of expression in lymphocytes have been reported^16^,^17^,^18^. CIB1 and CIB2 proteins have been identified in many cellular compartments, including cytoplasm, cell membranes, nucleus, and endoplasmic reticulum^14^,^19^,^20^. CIB1 can be N-myristoylated^14^,^20^, and therefore can localize to membranes either through association with integrins or by direct insertion.

The direct binding of CIB1 to αIIbβ3 can directly affect integrin function in platelets, possibly by inhibiting the binding of talin^21^. Nevertheless, CIB proteins can also associate with a bewildering variety of other partners, including numerous serine/threonine protein kinases (e.g., PAK1, FAK, DNA-PKcs), enzymes involved in the metabolism of second messengers (sphingosine kinase 1, Rac3), transmembrane ion channels (InsP3 receptor), and transcription factors (Pax3)^19^,^20^,^22^,^23^,^24^,^25^,^26^,^27^. Thus, it is not surprising that CIB proteins have been implicated in processes as diverse as, for example, cell survival and proliferation^26^, non-homologous end-joining DNA repair^25^, integrin signaling in skeletal muscle^12^, cytoskeleton and microtubule organization^28^, and macropinocytic cell entry of Kaposi’s sarcoma-associated herpesvirus^29^.

Given their wide intracellular distribution and polyvalent functions, it is difficult to dismiss *a priori* a potential role of CIB1 and CIB2 proteins at any step in the HIV-1 life cycle, and multiple distinct functions cannot be excluded. To palliate this uncertainty, we have undertaken a series of studies to better define the role of CIB1 and CIB2 in HIV-1 infection. The three main goals of this work were: i) to demonstrate that CIB1 and CIB2 play a role in HIV-1 replication not only in cell lines but also in human CD4+ T-lymphocytes, the most abundant natural target cells; ii) comprehensively evaluate the potential role of CIB proteins throughout the HIV-1 replication cycle, in order to identify the step(s) impaired by the downregulation of CIB1 and CIB2 expression; and iii) begin to characterize the mechanisms through which CIB proteins promote HIV-1 infection. We demonstrate that the knockdown of expression of both CIB1 and CIB2 impaired viral replication in target cells, including primary CD4+ T-lymphocytes. The action of CIB1 and CIB2 were specifically restricted to the early step of receptor-mediated viral entry.

In view of these findings, we further explored the consequences of the depletion of CIB proteins on the entry of HIV-1 into susceptible cells, a process dependent on a sequence of events involving different cellular partners. The initial adhesion of viral particles to the cell is mediated by relatively non-specific interactions with cell-surface molecules, including heparan-sulfate moieties, mannose receptors, and lectins^30^,^31^. These interactions facilitate the subsequent specific binding of the viral surface glycoprotein gp120 to CD4 expressed on surface of target cells, which triggers conformational changes in gp120 unmasking the binding site for the chemokine receptors CCR5 and/or CXCR4^32^,^33^. The use of these chemokine receptors defines virus tropism as R5-, X4- or dual-tropic. Binding to the chemokine receptors triggers further conformational changes in the viral envelope glycoprotein complex, leading to the fusion of the viral and cellular membranes – a process governed by the viral transmembrane glycoprotein gp41. HIV-1 infection of susceptible target cells can result either from interaction with viruses previously liberated from infected cells or via direct contact with infected cells^32^. Cell-to-cell transmission is an efficient process, facilitated by the accumulation of viral particles and receptors at the sites of cellular contacts. As for cell-free virus spread, however, productive infection of target cells by cell-to-cell transmission requires the assembly of virus particles competent for the fusion process and the expression of CD4 and co-receptors on the target cell^34^. We found that CIB1 and CIB2 did not influence the initial attachment of HIV-1 to target cells, but both were required for optimal CCR5- and CXCR4-dependent entry occurring through either cell-free or cell-to-cell transmission pathways. CIB1 and CIB2 were also required for optimal surface expression of several proteins previously implicated in viral entry, which may contribute, at least in part, to the ability of these CIB proteins to facilitate HIV-1 entry in natural target cells.

## Results

### Downmodulation of CIB1 and CIB2 in Jurkat T-cells

**Figure 1:**
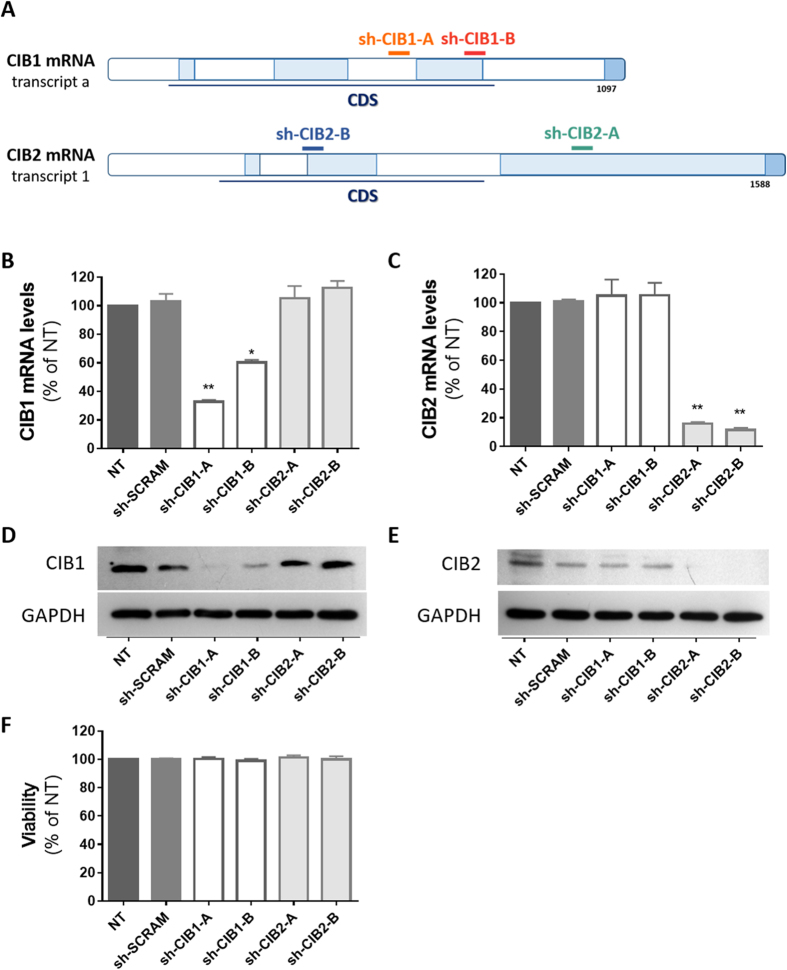
Knockdown of CIB1 and CIB2 in Jurkat T-cells. Jurkat cells were transduced by lentiviral vectors to express shRNAs targeting CIB1 or CIB2, and carrying a selectable marker (puromycin). After expansion and selection of shRNA-transduced cells, each population was evaluated for their knockdown efficiency and viability before challenge with HIV-1. (**A)** Structure of mRNA transcripts for CIB1 and CIB2 and shRNA target sites. Total RNA was extracted and cDNA was synthesized for quantification of CIB1, CIB2 and GAPDH mRNA levels. The ratio of CIB1/GAPDH mRNA (**B**) and CIB2/GAPDH mRNA (**C**) are expressed relative to those of non-transduced (NT) Jurkat cells. Knockdown at the protein level was confirmed by Western-blotting using antibodies recognizing CIB1 (clone 791119, R&D systems) (**D**), CIB2 (clone CIB2C12B11, Abcam) (**E**) and GAPDH (clone 6C5, Santa Cruz Biotechnology). Gels were run under the same experimental conditions and cropped images are shown (full-length blots are presented in Supplementary Figure S2). (**F**) After expansion and selection, the viability of shRNA-transduced cells was measured by Trypan blue exclusion. For panels B, C and F, values represent mean ± SEM of 4 or more independent transductions. *P < 0.05, **P < 0.01 versus sh-SCRAM (Wilcoxon paired test).

In initial studies, we sought to evaluate the extent that the infectivity of HIV-1 is impaired by reducing the expression of CIB1 and CIB2 in target cells. To do so, we transduced Jurkat T-cells with vectors resulting in the expression of shRNAs specifically targeting mRNA coding for these proteins through the RNA interference (RNAi) pathway, and selected cell populations stably expressing these shRNAs (Fig. 1). CIB1 mRNA levels were reduced by 70% and 40%, respectively, in cells expressing the sh-CIB1-A and sh-CIB1-B sequences (Fig. 1B); CIB2 mRNA levels were reduced by 80–90% in cells expressing both the sh-CIB2-A and sh-CIB2-B sequences (Fig. 1C). Despite the significant homology between CIB1 and CIB2, the knockdown of mRNA expression was highly specific, as expression of shRNAs against CIB1 had no significant effect on CIB2 mRNA levels, and conversely shRNAs against CIB2 had no significant effect on CIB1 mRNA levels. As a control, a scrambled sequence complementary to no human gene – sh-SCRAM – was used and had no effect on CIB1 and CIB2 mRNA levels. Western blotting also demonstrated that the knockdown of CIB1 and CIB2 mRNA levels led to similar reductions in protein expression, and confirmed the specificity of these effects (Fig. 1D,E). Stable expression of the shRNAs had no effect on cell viability (Fig. 1F), suggesting that normal levels of CIB1 and CIB2 expression are not required for T-cell viability.

### Reducing CIB1 and CIB2 expression impairs HIV-1 replication in Jurkat cells

**Figure 2:**
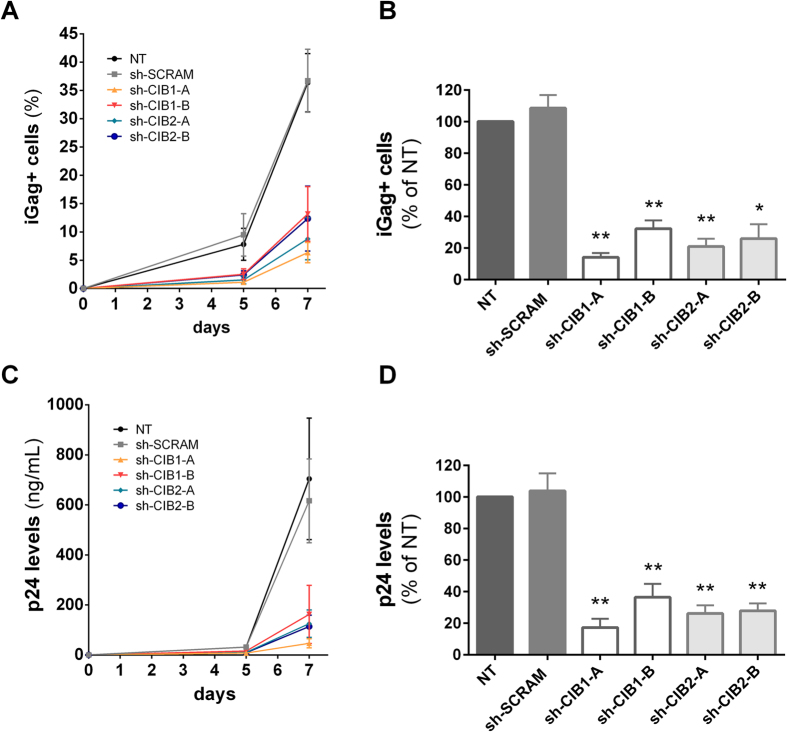
Depletion of CIB proteins impairs HIV-1 replication in Jurkat T-cells. Each shRNA population was infected with 2.5 ng of HIV-1_NL4-3_. On days 5 and 7 after infection, cells were stained with anti-Gag-PE antibody and analyzed by flow cytometry to quantify the percentage of intracellular Gag (iGag) positive cells (**A**). Viral replication was also assessed by quantifying HIV Gag levels in supernatant by p24 ELISA on days 5 and 7 of infection (**C**). The corresponding area under the curve (AUC) from replication kinetics is also shown (**B,D**). Values represent mean ± SEM of 4 or more independent transductions and are expressed relative to values obtained for non-transduced (NT) cells. *P < 0.05, **P < 0.01 versus sh-SCRAM (Mann-Whitney test).

To evaluate the effect of CIB1 and CIB2 downmodulation on HIV-1 replication, the Jurkat cell populations described above were infected with the X4-tropic HIV-1 strain NL4-3 (HIV-1_NL4-3_) and viral replication was measured on days 5 and 7 by quantifying both the percentage of viable cells expressing intracellular Gag (iGag) protein (Fig. 2A,B) and the amount of virus released into the supernatant, using a p24 ELISA test (Fig. 2C,D). Knockdown of both CIB1 and CIB2 strongly impaired viral replication. For cell populations with reduced CIB1 expression, the extent of impairment of viral replication reflected the extent of CIB1 knockdown. In cell lines expressing sh-CIB1-A and sh-CIB1-B sequences, both p24 levels and the percentage of Gag positive cells were reduced by 90% and 60%, respectively (Fig. 2). The two cell populations with reduced CIB2 expression displayed comparable reduction in viral infectivity (approximately 80% for both p24 levels and the percentage of Gag positive cells), consistent with the similar reduction in CIB2 expression observed in these cells. Taken together, these results indicate that both CIB1 and CIB2 play an important and non-redundant role in facilitating viral infection of Jurkat T-cells.

### Impairment of HIV-1 replication is specific to knockdown of CIB1 and CIB2

**Figure 3:**
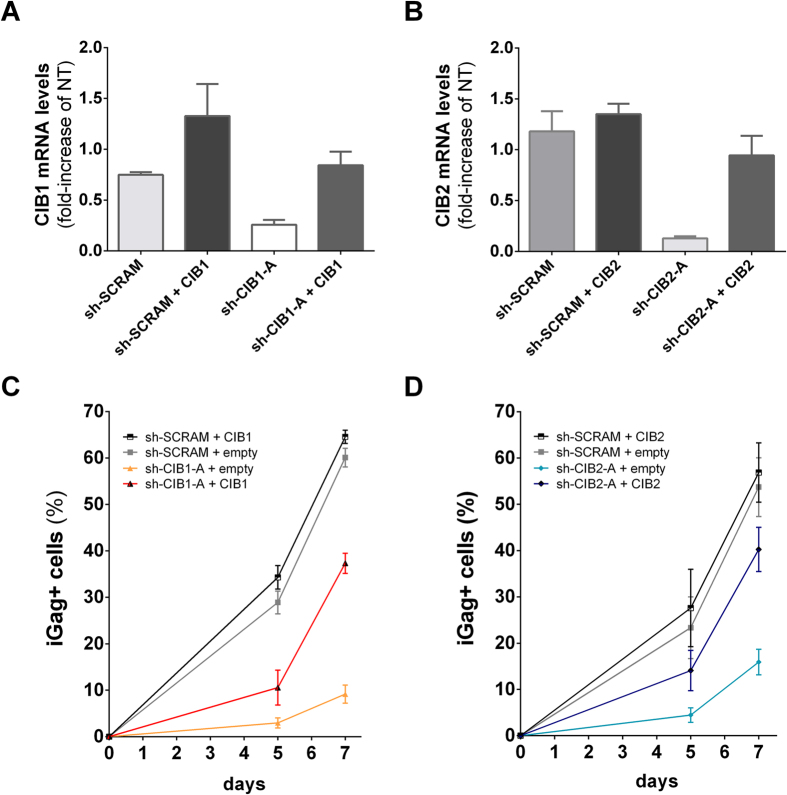
Impairment of HIV replication is specific to downmodulation of CIB proteins. To restore CIB protein levels in cells expressing shRNAs targeting CIB proteins, cells were co-transduced with viral vectors leading to the expression of shRNAs and viral vectors carrying RNAi-resistant cDNA for CIB1 or CIB2. Total RNA was extracted and cDNA was synthesized for quantification by quantitative real-time PCR. mRNA levels for CIB1 (**A**) and CIB2 (**B**) are relative to levels present in non-transduced (NT) Jurkat cells. Each co-transduced cell population was infected with 2.5 ng of HIV-1_NL4-3_, and viral replication was assessed by measuring the percentage of intracellular Gag (iGag) positive cells by flow cytometry on days 5 and 7 after infection. Results for CIB1-complemented cells (**C**) and CIB2-complemented cells (**D**) are shown. Values represent mean ± SEM of 3 or more independent transductions.

As shown above, knockdown of both CIB1 and CIB2 expression in Jurkat cells with shRNAs recognizing two distinct sequences on each gene reduced HIV-1 replication, arguing against the possibility that off-target effects of the shRNAs could be responsible for this phenotype. To firmly establish this point, we transduced cells stably expressing the shRNAs targeting CIB1 (sh-CIB1-A), CIB2 (sh-CIB2-A) and the control shRNA (sh-SCRAM) with vectors expressing either CIB1 or CIB2, but lacking the target sequence recognized by the shRNAs. The resistance to shRNA targeting was obtained either by introducing 6 silent mutations in the sequence targeted by the shRNA (CIB1) or deleting the 3’-untranslated region targeted by the shRNA (CIB2). As expected, transduction with expression vectors for CIB1 and CIB2 increased mRNA levels both in cells expressing sh-SCRAM and in those expressing shRNAs targeting the endogenous sequence (Fig. 3A,B). Increasing the expression of both CIB1 and CIB2 largely restored virus replication, despite the presence of the shRNAs targeting endogenous CIB1 and CIB2 (Fig. 3C,D).

### CIB1 and CIB2 knockdown impairs an early step in HIV-1 envelope-mediated viral entry

**Figure 4:**
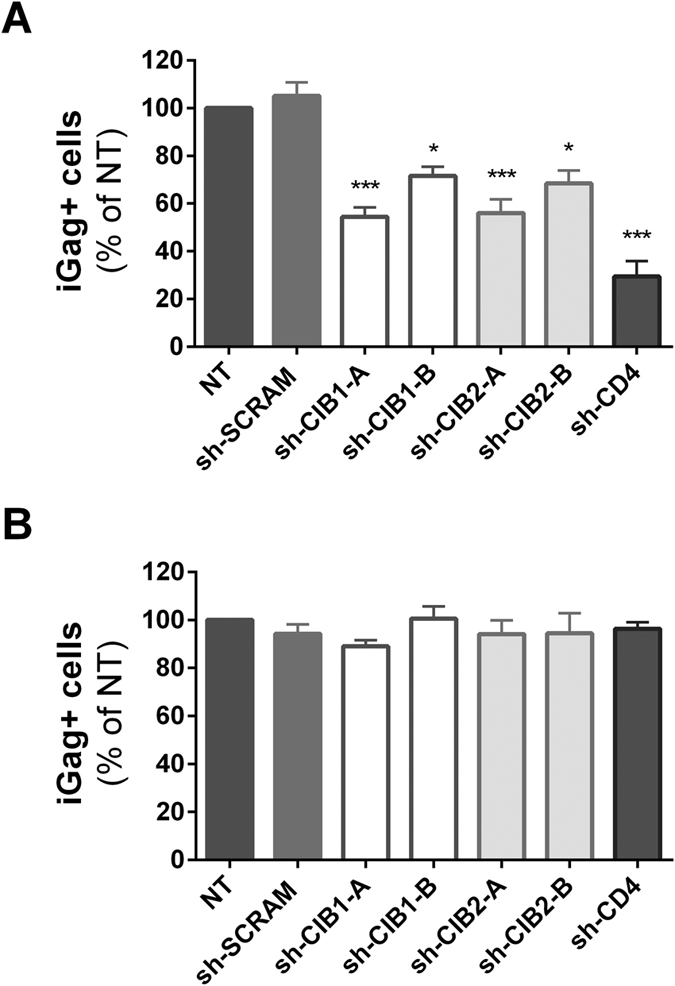
CIB1 and CIB2 are required for optimal HIV-1 envelope-mediated entry, but not VSV-G-mediated entry. Jurkat cells transduced with the indicated shRNAs were infected with viral particles pseudotyped with either HIV-1 NL4-3 (**A**) or VSV-G (**B**) envelopes, and 48 h later the percentage of intracellular Gag (iGag) positive cells was measured by flow cytometry. Values represent mean ± SEM of 5 or more independent transductions and are expressed relative to the percentage observed for non-transduced (NT) cells.*P < 0.05, ***P < 0.001 versus sh-SCRAM (Kruskal-Wallis test).

To begin to identify the step in the HIV-1 replication cycle that is impaired by downmodulation of CIB1 and CIB2, we infected Jurkat cells with envelope-defective pseudotyped viruses and measured the accumulation of intracellular Gag at 48 h, such that only the early steps of infection in a single cycle up to viral protein synthesis were evaluated. When cells were infected using NL4-3Δenv viruses pseudotyped with the NL4-3 HIV-1 envelope, viral infectivity in the different shRNA-transduced cells was comparable to that described above using intact HIV-1_NL4-3_ over multiple cycles. The infectivity of the pseudotyped viruses was significantly reduced in cells transduced with shRNAs targeting both CIB1 and CIB2 compared to that of non-transduced cells (NT) or those transduced with the control sh-SCRAM (Fig. 4A). As expected, cells transduced with a shRNA targeting CD4 also reduced the infectivity of viruses pseudotyped with the X4 HIV-1 envelope. Since Jurkat cells do not express CCR5, the requirement for CIB1 and CIB2 for virus entry mediated by an R5-tropic virus could not be tested in these cells.

Strikingly, when Jurkat cells were infected using NL4-3Δenv viruses pseudotyped with the VSV-G envelope, no discernable differences in infectivity were observed in cells in which CIB1 or CIB2 expression had been down-regulated by transduction with shRNAs (Fig. 4B). Taken together, these findings indicate that normal levels of CIB1 and CIB2 expression are required for optimal completion of early steps in the HIV-1 envelope-mediated entry process (e.g., attachment, receptor/co-receptor binding, membrane fusion). CIB1 and CIB2 do not appear to be required for subsequent transport of viral capsids to the nucleus, nuclear transport, integration, transcription and viral protein synthesis, steps that are shared by capsids entering the cytoplasm via both HIV-1 envelope and VSV-G envelope-mediated pathways.

### HIV-1 budding and particle infectivity are not impaired by depletion of CIBs

**Figure 5:**
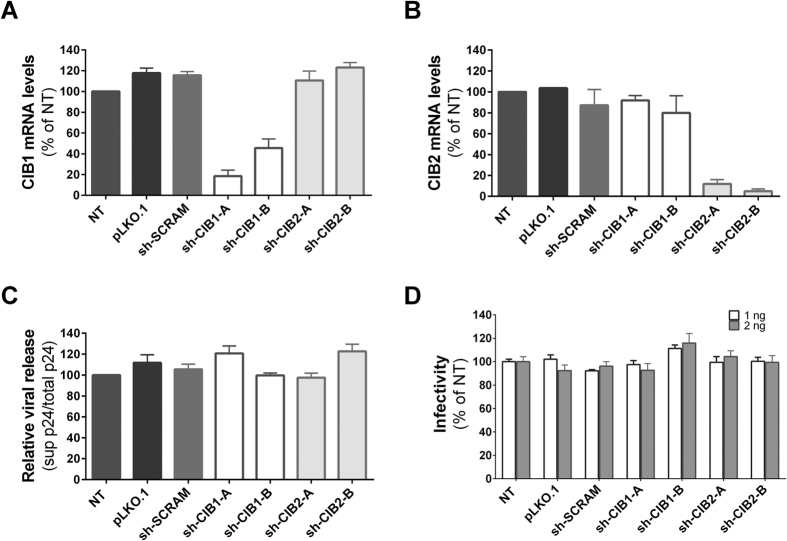
Downmodulation of CIB proteins does not impair late steps in the HIV-1 replication cycle. HeLa cells were transduced with lentiviral vectors inducing the expression of the indicated shRNAs and transduced cells were selected by culture in medium with increasing concentrations of puromycin. Total RNA was extracted and cDNA was synthesized for quantification by quantitative real-time PCR of mRNA levels for CIB1 (**A**) or CIB2 (**B**), as described in Fig. 1 legend. Results are expressed relative to those obtained for non-transduced (NT) HeLa cells. Values represent mean ± SEM of 2 independent transductions. (**C**) The transduced HeLa cells were transfected with 1 μg of pHIV-1_NL4-3_, and 24 h later p24 levels were quantified by ELISA in both cell supernatants and cell lysates. Relative viral release was calculated by dividing the amount of p24 in the supernatant by the total amount of p24 (supernatant + cell lysate). Values represent mean ± SEM of 3 independent transfections and are expressed relative results obtained for non-transduced (NT) cells. (**D**) Culture supernatants recovered from transfected HeLa populations containing the indicated amounts of p24 were used to infect HeLaP4C5 cells, which carry the β-galactosidase gene under the control of the HIV-1 LTR. Tat-inducible β-galactosidase activity was quantified by colorimetric measurement of metabolized CPRG substrate. Values represent mean ± SEM of 3 independent infections, each done in triplicate and are expressed relative to results obtained for non-transduced (NT) cells.

Since CIB proteins can localize at the plasma membrane, we analyzed whether CIB1 and CIB2 are also required for viral late steps. To do so, HeLa cells were transduced with vectors resulting in the expression of shRNAs targeting CIB1 and CIB2 or control vectors, and then transfected with pHIV-1_NL4-3_. Virus budding and release were assessed 24 h later by determining the proportion of p24 released into the medium. As expected, CIB1 and CIB2 mRNA expression in HeLa cells was reduced in cells transduced with vectors expressing shRNA specifically targeting the sequence, but not in cells transduced with vectors expressing shRNA targeting the paralogous sequence, the control shRNA (sh-SCRAM), or the empty vector (pLKO.1) (Fig. 5A,B). Despite the efficient knockdown of CIB1 and CIB2 mRNA obtained by shRNA, no impairment of viral release by these cells was observed (Fig. 5C). We confirmed that this assay could detect impairment in viral release by transfecting non-transduced HeLa cells with pNL4-3ΔVpu. Due to the absence of Vpu, the unopposed activity of tetherin expressed by HeLa cells reduced viral release by 70% compared to that of cells transfected with wild-type pNL4-3 (data not shown).

We also evaluated the possibility that downmodulation of CIB1 or CIB2 could influence the infectivity of the viral particles released. To do so, a fixed amount of virus (1 or 2 ng p24) obtained from the supernatant of the transfected HeLa cells was used to infect the P4C5 reporter cell line, which expresses the β-galactosidase under the control of HIV-1 LTR. As shown in Fig. 5D, the infectivity of viruses obtained from cells expressing normal or reduced levels of CIB1 and CIB2 were not significantly different. Thus, reduction of CIB1 and CIB2 expression did not influence either the efficiency of viral release or the infectivity of the released viral particles.

### Downmodulation of CIB1 and CIB2 impair HIV-1 replication in primary CD4+ T-lymphocytes

**Figure 6:**
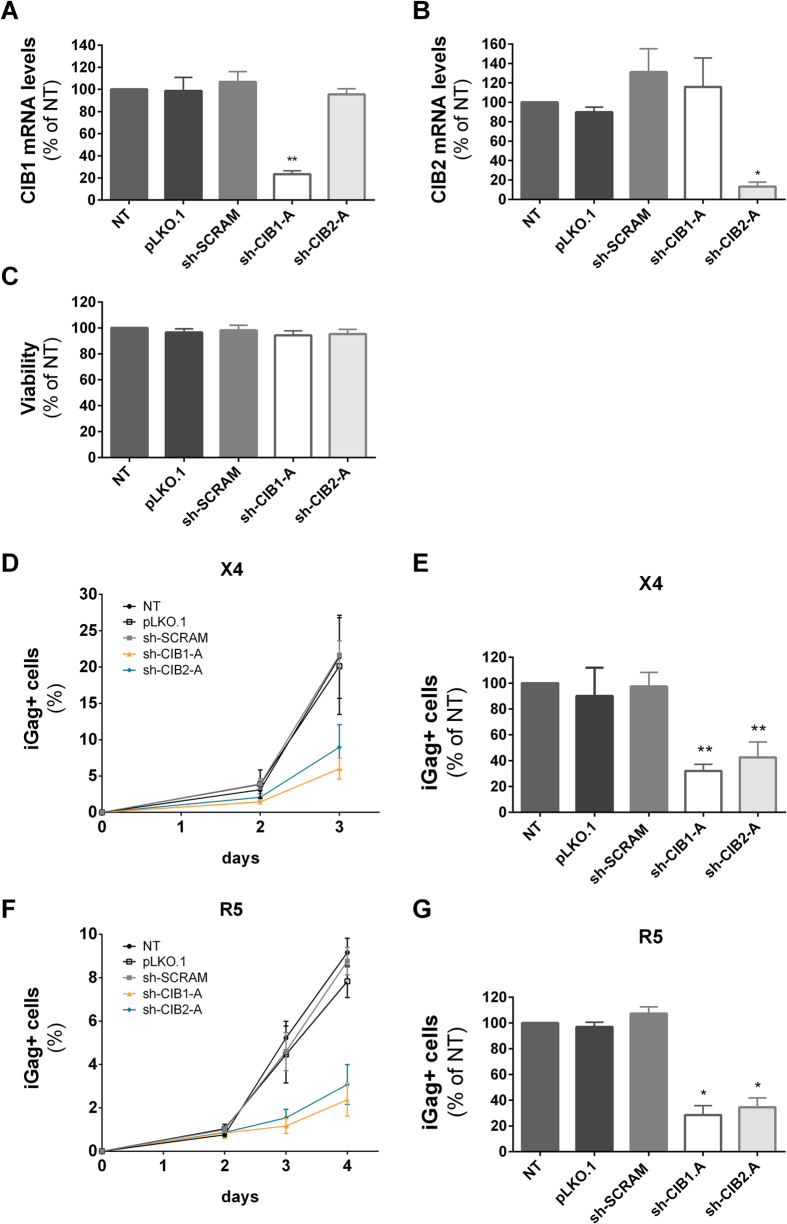
Knockdown of CIB proteins affects HIV-1 replication in primary CD4+ T-lymphocytes. Activated CD4+ T-lymphocytes were transduced with the empty vector (pLKO.1) or vectors leading to the expression of the indicated shRNAs, and transduced cells were selected and expanded. Total RNA was extracted and cDNA was synthesized for quantification by quantitative real-time PCR of mRNA levels for CIB1 (**A**) and CIB2 (**B**). Results are expressed relative to values obtained for non-transduced (NT) primary CD4+ T-lymphocytes after normalization to GAPDH. (**C**) The viability of the transduced cells after expansion was evaluated using the MTT test. Each transduced cell population was infected with 10 ng of HIV-1_NL4-3_ (**D**,**E**) or with 80 ng of HIV-1_NLAD8_ (**F**,**G**), and on the indicated days after infection viral replication was assessed by determining the percentage of intracellular Gag (iGag) positive cells by flow cytometry. The replication kinetics of HIV-1 in the indicated cell populations (**D,F**) and the area under the curve (AUC) derived from the replication kinetics (**E,G**) are shown. The values represent mean ± SEM of 4 or more independent transductions and are expressed relative to those obtained for non-transduced (NT) cells. *P < 0.05; **P < 0.01 versus sh-SCRAM (Mann-Whitney test).

To evaluate the impact of down modulation of CIB1 and CIB2 on viral replication in natural host target cells, primary human CD4+ T-cells were purified from peripheral blood, activated with anti-CD3/CD28 beads, and transduced with lentiviral vectors allowing the expression of the most efficient shRNAs targeting CIB1 and CIB2. The extent and specificity of the reduction of mRNA expression in these populations was quite similar to that observed in Jurkat and HeLa cells (Fig. 6A,B), and transduction of CD4+ T-cells did not affect cell viability (Fig. 6C) or cell proliferation (data not shown). The baseline expression level of CIB1 and CIB2 mRNAs relative to GAPDH mRNA was noted to be somewhat lower in primary CD4+ T-cells than in the cell lines (data not shown).

Subsequently, the transduced CD4+ T-cells were infected with either the X4-tropic HIV-1_NL4-3_ or the R5-tropic HIV-1_NLAD8_ and viral replication was evaluated over 4 days. Both HIV-1 strains replicated with significantly lower efficiency in CIB-depleted CD4+ T-cells compared to that observed in cells transduced with the control shRNA (sh-SCRAM), the empty vector (pLKO.1) and in non-transduced cells (Fig. 6D–G). The percentage of cells expressing intracellular Gag at day 3 post-infection with HIV-1_NL4-3_ was decreased by 70% and 60%, respectively, following down modulation of CIB1 and CIB2 (Fig. 6D,E). A similar reduction was observed when shRNA-transduced populations were challenged with HIV-1_NLAD8_ (Fig. 6F,G). Therefore, CIB1 and CIB2 are required for replication of both R5- and X4-tropic HIV-1 strains, in their natural target cells.

### CIB1 and CIB2 knockdown in primary CD4+ T-cells impairs an early step in HIV-1 replication

**Figure 7:**
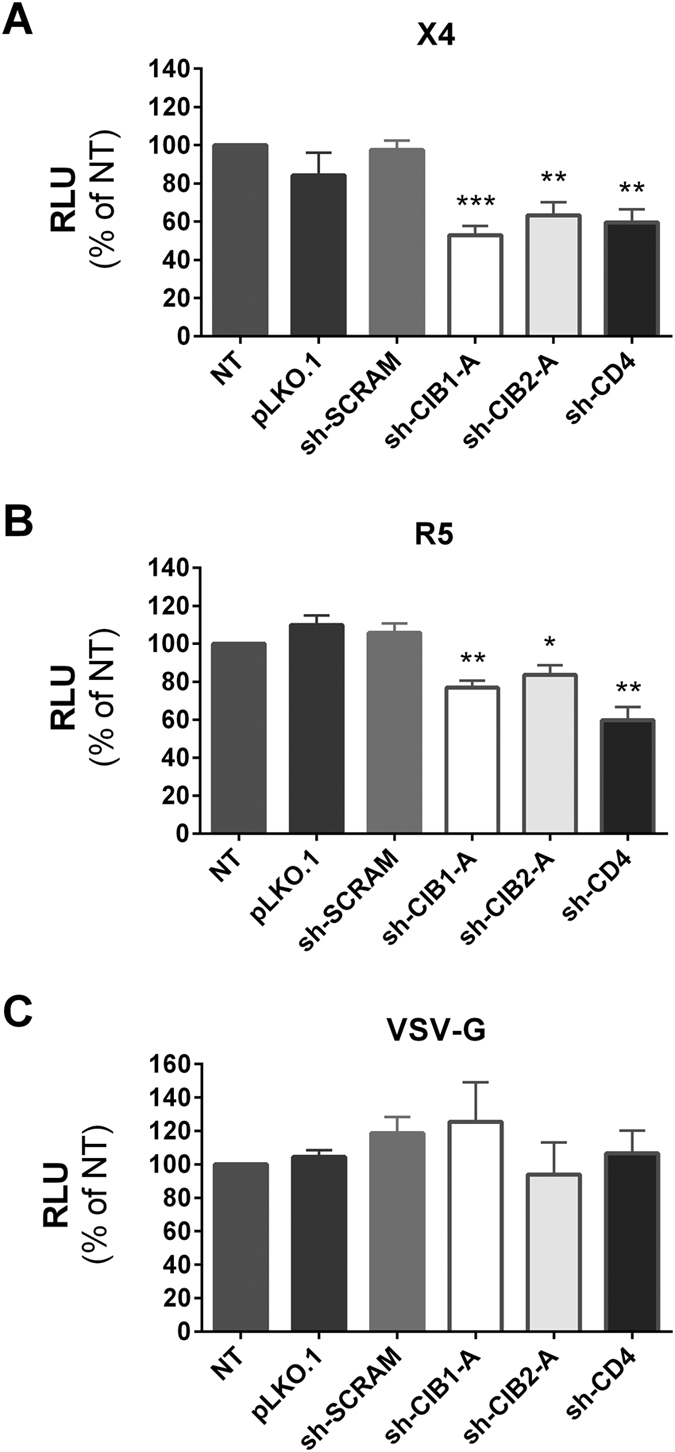
Env-dependent effect of CIB proteins knockdown on HIV-1 infection of primary cells. Activated CD4+ T-lymphocytes transduced with the empty vector (pLKO.1) or vectors leading to the expression of the indicated shRNAs were infected with HIV-1 particles carrying the luciferase gene in place of *nef* and pseudotyped with X4-tropic HIV-1 Env (**A**), R5-tropic HIV-1 Env (**B**), or VSV-G (**C**). Luciferase activity in target cells was measured 48 h after infection. Values represent mean ± SEM of 5 or more independent transductions and are expressed relative to luciferase activity observed in non-transduced (NT) cells. *P < 0.05, **P < 0.01, and ***P < 0.001 versus sh-SCRAM (Mann-Whitney test).

We conducted single-cycle infectivity assays to verify that CIB1 and CIB2 knockdown in CD4+ T-cells impairs an early step in HIV-1 envelope-mediated viral entry, similar to that observed in Jurkat cells. To this end, shRNA-transduced CD4+ T-cells were infected with viral particles produced by cells transfected with envelope-defective HIV-1 viruses carrying the luciferase gene in the place of *nef* and which had been pseudotyped with either a X4-tropic HIV-1 envelope (NL4-3), a R5-tropic HIV-1 envelope (AD8), or the VSV-G envelope glycoprotein, and viral infectivity was measured by determining luciferase activity in target cells. As in Jurkat cells, CIB1- and CIB2-depleted CD4+ T-lymphocytes displayed a significant decrease in susceptibility to infection when challenged with HIV-1-enveloped particles but not with VSV-G-enveloped particles (Fig. 7). Importantly, as observed in multiple cycle experiments, the infectivity of viruses pseudotyped with an R5-tropic HIV-1 envelope was also significantly reduced in cells transduced with shRNAs targeting both CIB1 and CIB2 (Fig. 7B). The extent of this reduction, however, was smaller than that observed for viruses pseudotyped with the X4-tropic HIV-1 envelope (Fig. 7A). In contrast, the infectivity of viruses pseudotyped with both R5-tropic and X4-tropic HIV-1 envelopes were impaired to a similar extent in cells transduced with a shRNA targeting CD4. These results show that HIV-1 entry process in CD4+ T-cells mediated by both CXCR4 and CCR5 co-receptors is reduced following the knockdown of CIB1 and CIB2, whereas VSV-G-mediated entry is independent of CIB1 and CIB2 expression (Fig. 7C).

### CIB1 and CIB2 have a role in post-attachment steps in primary CD4+ T-cells

**Figure 8:**
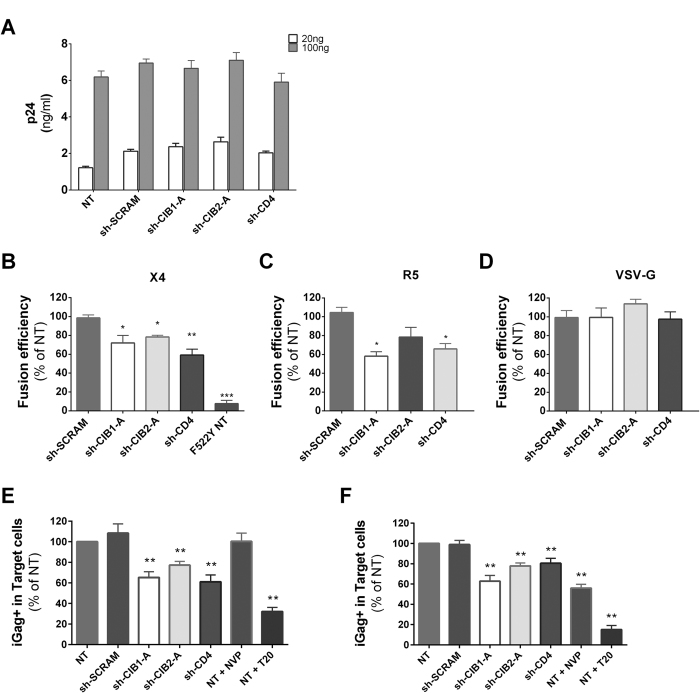
Depletion of CIB proteins in primary CD4+ T-lymphocytes influences Env-mediated virus entry but not viral attachment. **(A)** Viral binding assay. Activated CD4+ T-lymphocytes transduced with vectors leading to the expression of the indicated shRNAs were incubated with the indicated amounts of HIV-1_NL43_ (20 or 100 ng p24) in serum-free RPMI-1640 medium for 2 h at 4 °C. Cells were washed to remove unbound virus, pelleted, and lysed, after which cell-associated HIV p24 was measured by ELISA. Values represent mean ± SEM of 4 independent transductions. **(B–D**) Viral fusion and entry. CD4+ T-lymphocytes expressing the indicated shRNAs were exposed to HIV-1 particles (200 ng p24) containing BlaM-Vpr and pseudotyped with either an X4-tropic HIV-1 Env (**B**), R5-tropic HIV-1 Env (**C**), or VSV-G (**D**) for 4 hours, after which the CCF2 substrate was added. Cells containing the cleaved substrate were quantified by flow cytometry to assess viral entry. As a control, non-transduced cells were infected with a virus expressing a HIV-1 Env with the F522Y mutation, which prevents viral entry (F522Y NT in panel B). Values represent the mean ± SEM of at least 4 independent transductions and are expressed relative to results obtained for non-transduced (NT) cells. *P < 0.05, **P < 0.01, ***P < 0.001 versus sh-SCRAM (Mann Whitney test). **(E**,**F)** Cell-mediated virus transmission. Non-transduced activated primary CD4+ T-lymphocytes previously infected using 100 to 500 ng of HIV-1_NL43_ and labeled with CFSE dye were used as donor cells. Untransduced cells and those transduced with the indicated shRNAs were co-cultured with the CFSE-labeled donor cells, such that targets could be distinguished from donors. The appearance of intracellular Gag positive (iGag+) target cells was measured 4 h (**E**) or 24 h (**F**) later, to quantify the efficiency of virus transfer and productive infection, respectively. To serve as controls, untransduced cells were treated with either nevirapine (NVP) or T-20. Values represent the mean ± SEM of 3 independent transductions and are expressed relative to the values observed for non-transduced cells. *P < 0.05, **P < 0.01 versus sh-SCRAM (Mann-Whitney test).

To better identify the step in viral entry that is facilitated by CIB1 and CIB2, we first assessed if viral attachment was affected by downmodulation of CIB1 and CIB2 expression. To achieve this, activated CD4+ T-cells that had been transduced with expression vectors for shRNAs were incubated with virus-containing supernatant for 2 h at 4 °C to prevent entry, washed and the amount of virus bound to cells was quantified by p24 ELISA. As shown in Fig. 8A, the amount of virus bound to CD4+ T-cells was not reduced in cells in which CIB1 or CIB2 expression had been downmodulated, indicating that they do not play a role in virus adhesion, a process largely independent of virus receptors. Next, we evaluated whether CIB1 and/or CIB2 are involved in virus-cell fusion by challenging activated CD4+ T-cells with HIV-1_NL4-3_ particles carrying β-lactamase-Vpr chimeric proteins (BlaM-Vpr) and pseudotyped with either X4-tropic or R5-tropic HIV-1 envelopes or the VSV-G envelope. As a result of virion fusion, BlaM-Vpr is delivered into the cytoplasm of target cells and this transfer can be detected by the change in color of the CCF2 dye resulting from its cleavage by β-lactamase^35^. For activated CD4+ T-cells in which CIB1, CIB2 and CD4 had been downmodulated, viral entry was significantly reduced, but only when infected with viruses pseudotyped with HIV-1 envelopes, not when exposed to viruses pseudotyped with VSV-G (Fig. 8B–D). Thus, reduced expression of both CIB1 and CIB2 impair the cell-free viral entry mediated by HIV-1 envelope-induced fusion.

The efficiency of HIV-1 infection can be improved by cell-to-cell transmission, raising the possibility that cell-to-cell transmission would abrogate the defect in infectivity seen following downmodulation of CIB1 and CIB2 expression. To evaluate this question, activated CD4+ T-cells (target cells) were cultured with NL4-3-infected CD4+ T-cells that had been labeled with the cytosolic dye CFSE (donor cells), allowing the identification of target and donor cells in the culture by FACS analysis. The percentage of Gag + cells in the target cell populations was determined at 4 h to detect virus particle transfer events (Fig. 8E), and after 24 h of co-culture to measure productive infection events (Fig. 8F). At 4 h of co-culture, the percentage of intracellular Gag-positive cells was significantly lower in target cells in which CIB1, CIB2 and CD4 expression had been downmodulated, demonstrating a reduced transfer of viral particles from donor cells. In our assay, the vast majority of the intracellular Gag detected in target cells results from the fusion of the viral and cellular membranes^34^. This conclusion is supported by the fact that the percentage of intracellular Gag + cells is not perturbed by NVP treatment at this time, but is dramatically reduced by preventing membrane fusion by T20 (Fig. 8E, lanes NT + NVP and NT + T20). Reducing the expression of both CIB1 and CIB2 also significantly decreased productive infection efficiency measured at 24 h (Fig. 8F), showing that CIB1 and CIB2 are required for both cell-free and cell-to-cell virus transmission.

### Effect of the knockdown of CIB1 and CIB2 on the surface expression of molecules implicated in HIV-1 entry

**Figure 9:**
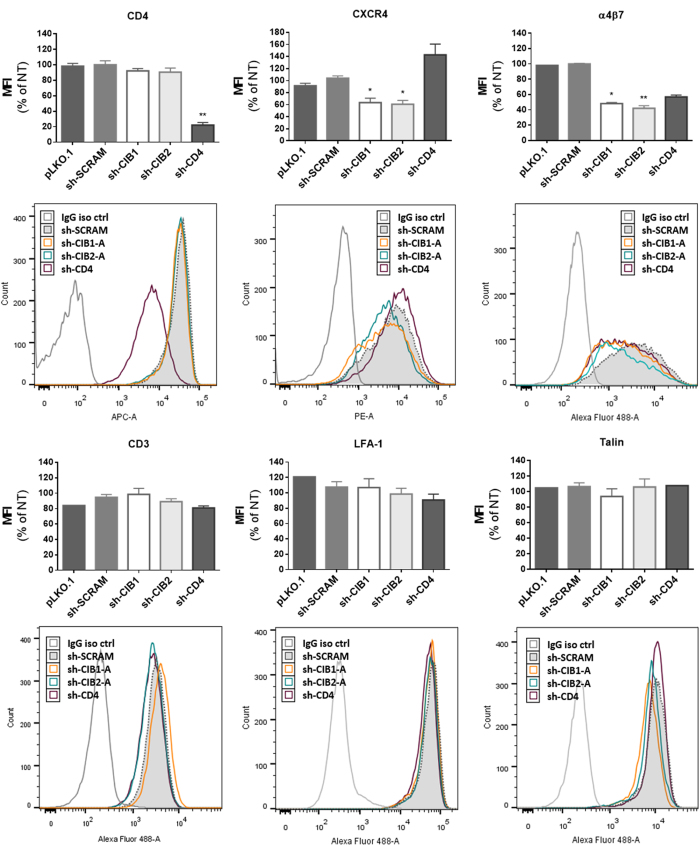
Effect of downmodulation of CD4 and CIB proteins in CD4+ T-lymphocytes on the expression of proteins implicated in HIV-1 entry. Activated CD4+ T-lymphocytes were transduced with the empty vector (pLKO.1) or vectors leading to the expression of the indicated shRNAs, and the surface expression of the following molecules was measured by flow cytometry: CD3, CD4, CXCR4, LFA-1 and α4β7. Intracellular talin expression was also evaluated in permeabilized cells. In each case, the mean fluorescence intensity (MFI) and a representative fluorescence histogram are shown. For MFI, the values represent the mean ± SEM of 4 independent experiments using two different shRNAs per targeted gene and are expressed relative to values obtained for non-transduced cells. *P < 0.05, **P < 0.01 versus sh-SCRAM (Kruskal-Wallis test).

Impaired expression at the cell surface of molecules necessary for HIV-1 entry is a possible mechanism accounting for the reduced viral entry seen following downmodulation of CIB1 and CIB2. To begin to evaluate this possibility, we measured cell surface expression of CD3, CD4, CXCR4, LFA-1 and α4β7, as well as the intracellular molecule talin on activated CD4+ T-lymphocytes by flow cytometry (Fig. 9). CD4 surface expression was potently downmodulated only in the cell population transduced by shRNA targeting CD4, demonstrating that downmodulation of CIB1 and CIB2 does not modulate CD4 expression. CXCR4, the co-receptor for X4-tropic strains such as HIV-1_NL4-3_, was significantly reduced in cells in which either CIB1 or CIB2 had been downmodulated, showing an impact of these proteins on the surface expression of this chemokine receptor.

The integrins LFA-1 (αLβ2) and α4β7 have been shown to facilitate both cell-free and cell-to-cell virus entry^36^,^37^,^38^. In the latter situation, LFA-1 clusters in virological synapses along with cytoskeletal protein talin^33^. The surface expression of α4β7 integrin was significantly decreased in cells in which CIB1 and CIB2 had been downmodulated. Downmodulation of CIB1 and CIB2 expression had no effect on the surface expression of talin, LFA-1 and CD3.

**Figure 10:**
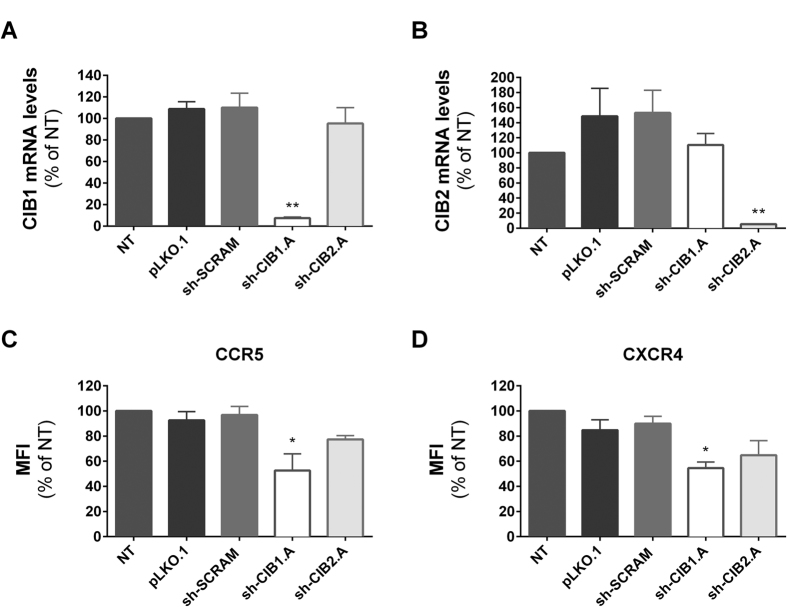
Effect of downmodulation of CIB proteins in PM1 cells on the expression of HIV-1 co-receptors. PM1 cells were transduced with the empty vector (pLKO.1) or vectors leading to the expression of the indicated shRNAs. Total RNA was extracted and cDNA was synthesized for quantification of CIB1, CIB2 and GAPDH mRNA levels. The ratio of CIB1/GAPDH mRNA (**A**) and CIB2/GAPDH mRNA (**B**) are expressed relative to those of non-transduced (NT) PM1 cells. The surface expression of CCR5 (**C**) and CXCR4 (**D**) was measured by flow cytometry. The mean fluorescence intensity (MFI) values represent the mean ± SEM of 3 independent experiments and are expressed relative to values obtained for non-transduced cells. *P < 0.05, **P < 0.01 versus sh-SCRAM (One-way ANOVA).

Primary CD4+ T-lymphocytes activated *in vitro* express very low levels of CCR5, the co-receptor for R5-tropic strains, which precluded evaluating the effect on CIB1 and CIB2 knockdown on the expression of this co-receptor in primary cells. Therefore, we assessed surface expression in PM1 cells, a T-cell line that expresses CCR5 constitutively. As shown in Fig. 10, CIB1 and CIB2 mRNA expression in PM1 cells was potently reduced upon transduction with vectors expressing shRNA specifically targeting the sequence (Fig. 10A,B). Also, knockdown of CIB1 and CIB2 led to comparable reductions in the surface expression of CCR5 and CXCR4 (Fig. 10C,D). In this cell type, greater reductions in expression of both co-receptors were observed following knockdown of CIB1 than knockdown of CIB2, and only the changes observed after CIB1 knockdown achieved statistical significance in this small series of experiments.

These results indicate that reduced expression of CIB1 and CIB2 is associated with a decrease in the expression of some, but not all, cellular surface molecules required for the early steps of HIV-1 replication, effects that may contribute to the impairment of viral infectivity observed following downmodulation of CIB1 and CIB2.

## Discussion

In this study, we have extensively characterized the impact of downmodulating the expression of CIB1 and CIB2 on the infectivity of HIV-1, and begun to evaluate the mechanisms explaining the requirement of these proteins for optimal viral replication. As previously observed for CIB2, we found that knockdown of CIB1 impaired HIV-1 replication in Jurkat cells, and demonstrated for the first time that both of these proteins are also required for optimal viral replication in primary human CD4+ T-lymphocytes. Knockdown of both CIB1 and CIB2 impaired an early step in receptor-mediated viral entry, and both cell-free and cell-mediated viral infections were affected. In contrast, we found no evidence that the level of CIB1 and CIB2 expression influenced cell viability, cell proliferation, receptor-independent viral binding to the cell surface, later steps involved in the transport and uncoating of the viral capsid, nuclear import and integration, or the production, export and infectivity of progeny virions. CIB1 and CIB2 knockdown were shown to reduce the expression of surface receptors implicated in HIV-1 infection, suggesting at least one mechanism through which these proteins promote viral infection.

An interesting observation in our study was the finding that knockdown of both CIB1 and CIB2 impaired viral infectivity, and no striking differences were observed for any of the parameters evaluated in our study comparing CIB1 and CIB2 knockdown cells. The extent that the functions of CIB1 and CIB2 are overlapping or distinct is incompletely understood. CIB1 regulates the activation of αIIbβ3 integrin in platelets^39^, but in a CIB1 knockout murine model, abnormal activation of platelets was not observed, possibly reflecting compensatory overexpression of other CIB proteins in megakaryocytes^18^, but in other cell types, the deleterious effects of CIB1 knockdown were not compensated^40^. Our findings indicate, however, that CIB1 and CIB2 were not redundant in Jurkat cells and CD4+ T-lymphocytes, because knockdown of either of these proteins impaired HIV-1 infectivity. This could reflect either the inability of these cells to compensate for the loss of one CIB by the increased expression of a homolog and/or specific differences in the function of these related proteins. For example, the proteins could participate in the same cellular function (e.g., as components of a multi-protein complex or participants in different steps of the same pathway), and in this case it would be expected that knockdown of either protein would produce generally similar phenotypes.

Our results indicate that changes in CIB1 and CIB2 expression did not modify several other cellular properties that could impact viral replication. Recent studies have shown that CIB1 can prevent death of cancer cells^41^, at least in part by activating kinases necessary for survival^42^. We found, however, that the viability of Jurkat cells and activated CD4+ T-lymphocytes either before or after HIV-1 infection was not influenced by CIB1 and CIB2 knockdown. Similarly, reduced expression of these proteins did not influence the receptor-independent adherence of viruses to the cell surface.

A key finding in our study was the observation that knockdown of CIB1 and CIB2 had no impact on the infectivity of HIV-1 virions pseudotyped with the VSV-G envelope in either Jurkat cells or CD4+ T-lymphocytes. VSV-G-mediated entry delivers the capsid deep into the cytoplasm. The subsequent events, including uncoating, nuclear entry, integration, expression of viral proteins, assembly and transport of progeny virions, follow the same pathways used by viruses whose entry is mediated by the HIV-1 envelope. In this regard, CIB1 and, to a lesser extent, CIB2 have been shown in various cell models to participate in the stimulation of downstream signaling pathways that could influence these events. For example, CIB1 can promote activation of the MAPK/ERK pathway^23^, which, in turn, stimulates the activation of AP-1 and NF-κB transcription factors. Nevertheless, our findings suggest that neither CIB1 nor CIB2 play a determinant role in the regulation of these later steps in the HIV-1 replicative cycle.

Taken together, our results indicate that the effects of CIB1 and CIB2 knockdown specifically affect early events in virus infection, encompassing receptor-mediated viral binding, membrane fusion, and possibly, early post-fusion modification of the cellular cytoskeleton. In this context, we observed that CIB1 and CIB2 knockdown resulted in reduced intensity of surface expression on activated CD4+ T-lymphocytes of CXCR4 co-receptors and α4β7 integrins, but not CD4, and CIB1 knockdown reduced the expression of CCR5 in PM1 cells. Several reports have indicated that changes in CXCR4 expression of the magnitude observed in our studies reduce the infectivity of X4-tropic HIV-1^43^,^44^, and relatively small changes in CCR5 expression have also been reported to reduce cellular susceptibility to R5-tropic HIV-1^43^,^45^,^46^. Similarly, the HIV-1 envelope is known to engage activated α4β7, and the level of expression of this integrin on CD4+ T-lymphocytes has been shown to influence their sensitivity to HIV-1 infection^47^. Thus, decreased surface expression of key cellular proteins interacting with gp120 resulting from CIB1 and CIB2 knockdown could contribute to the reduced sensitivity to HIV-1 infection observed in our study, although our results neither establish this as the mechanism, nor exclude the contribution of other processes.

Indeed, currently available information concerning the function of CIB proteins strongly suggests that their mode of action in promoting HIV-1 infection will ultimately be found to also involve intensification of out-to-in signaling events occurring at the cell membrane following virus/receptor interactions. CIB proteins can localize to membranes, either through the direct insertion of N-terminal myristolated forms or through their interaction with integrins, and, in several examples, have been shown both to directly modify integrin function and/or serve as adaptor-like molecules that concentrate associated signaling proteins, sometimes in a calcium-dependent fashion, to the sites of their attachment, resulting in important functional consequences. Following virus binding to target cells, the HIV-1 envelope is known to induce a variety of signaling events, including CD4-dependent mobilization of the Lck tyrosine kinase^48^,^49^, CXCR4- and CCR5-dependent signaling events such as CXCR4-dependent activation of NFAT^47^,^50^,^51^,^52^, and α4β7-induced activation of αLβ2 integrins (LFA-1)^38^. The enhancement of these signaling cascades by recruitment of CIB1 and CIB2 proteins and their associated proteins could promote the formation and stabilization of virological synapses, contribute to modifications in membrane structure favoring fusion with the viral envelope, and/or participate in the reorganization of the cytoskeletal network necessary for capsid entry and transport. Indeed, even prior to viral attachment, CIB proteins may modulate signals emanating from co-receptors, modifying their internalization and/or recycling, thereby accounting for the reduced expression of CXCR4, CCR5 and α4β7 observed in our studies.

Considerable further work is required to directly demonstrate the localization of CIB1 and CIB2 on lymphocyte membranes, determine the mechanisms responsible for their distribution, identify the putative interacting partners present, and explore their impact on viral entry. In undertaking these studies, it is important to recognize that their importance may depend on both the tropism of the incoming virus and the activation status of the target cell. In activated T-cells, gp120-induced signaling through CCR5 or CXCR4 has been found to improve infectivity in some but not all studies (reviewed in^53^,^54^,^55^). In resting T-cells, however, where cortical actin serves as a barrier to productive infection, viral induced chemokine receptor signaling becomes obligatory for the establishment of latently infected cells^56^.

In conclusion, our work demonstrates that knockdown of both CIB1 and CIB2 impaired an early step in receptor-mediated viral entry involved in both cell-free and cell-mediated viral infection, and was associated with reduced surface expression of CXCR4, CCR5 and α4β7. Elucidation of the mechanisms through which CIB1 and CIB2 promote infection is likely to provide new insights into the strategies used by HIV-1 to co-opt normal cell functions to efficiently transfer capsids into the cytosol.

## Material and Methods

### Cells

HEK 293 T cells (ATCC) and HeLa cells (ATCC) were maintained in DMEM medium supplemented with 10% heat-inactivated fetal bovine serum (FBS, Gibco, Life Tecnologies), penicillin G (100IU/ml) and streptomycin (100 μg/ml). P4C5 cells^57^ were cultured in supplemented DMEM containing G418 (500 μg/ml) and hygromycin B (100 μg/ml). Jurkat clone E6-1 T-cells (ATCC) and PM1 cells^58^ were grown in RPMI-1640 medium supplemented with 10% FBS, penicillin G (100IU/ml), streptomycin (100 μg/ml) and amphotericin B (0.25 μg/ml). PBMCs from healthy donors were isolated by density gradient centrifugation using SepMate tubes (StemCell Technologies). Primary CD4+ T-cells were then purified by negative selection (EasySep Human CD4+ T Cell Enrichment Kit, StemCell Techonologies), according to the manufacturer’s instructions. Primary CD4+ T-cells were cultured in RPMI medium supplemented with 10% FBS and IL-2 (100IU/ml), and activated with Dynabeads anti-CD3/CD28 microspheres (Life Technologies). Anonymized peripheral blood samples were obtained from healthy donors through the Etablissement Français du Sang in accordance with the approved guidelines, and informed consent was obtained from all donors. All experimental protocols were approved by the Conseil d’Unité of Inserm U941, and conducted according to approved guidelines from the Institut Universitaire d’Hématologie. All cultures were maintained at 37 °C in a humidified atmosphere with 5% CO_2_.

### Production and transduction of lentiviral vectors expressing shRNA

To express short hairpin RNA (shRNA) in target cells, we first produced lentiviral vectors by co-transfecting HEK 293 T cells with pLKO.1-Puro-shRNA constructs (see below), the R8.74 packaging plasmid (Addgene) and a vesicular stomatitis virus glycoprotein (VSV-G) envelope plasmid^59^. Cells were transfected using jetPEI transfection reagent (Polyplus Transfection). The pLKO.1-shRNA lentiviral knockdown system has been described previously^4^. The two most efficient shRNAs for CIB1 and CIB2 in terms of knockdown profile were used from a previous RNAi lentiviral library (contribution from Dr. Luís Moita) to transduce target cells (see Supplementary Table S1). As controls, we used a pLKO.1-empty vector (not expressing shRNA), a pLKO.1-sh-SCRAM vector, expressing a scrambled sequence complementary to no human gene, and a shRNA targeting mRNA coding for CD4 (Open Biosystems, Ref. TRCN0000057616). All viral supernatants were collected 2 days post-transfection, clarified by centrifugation, aliquoted and stored at −80 °C, and then quantified by p24 ELISA (Innogenetics) prior to use.

Different cell types were transduced using the lentiviral vectors described above to express the respective shRNA. To transduce Jurkat E6-1 cells, one million cells were exposed to 50 ng of lentiviral vectors expressing each shRNA for 2 h at 37 °C, and transduced cells were selected by gradually increasing concentrations of puromycin until a concentration of 2 μg/ml was reached. For HeLa cells, 25 ng of each shRNA lentiviral vector was used to transduce 1 × 10^5^ cells for 2 h at 37 °C. Twenty-four hours post-transduction, cells were selected by gradually increasing concentrations of puromycin until a concentration of 1 μg/ml was reached.

For primary CD4+ T-cells, 100 ng of each shRNA lentiviral vector was used to transduce 1 × 10^6^ anti-CD3/CD28-stimulated CD4+ T-cells for 2 h at 37 °C. Twenty-four hours after transduction, cells were selected by gradually increasing concentrations of puromycin until a concentration of 2 μg/ml was reached. All shRNA-transduced cells underwent selection without cloning, thus these populations were polyclonal in all experiments.

### Cell viability and measurement of mRNA expression level in shRNA transduced cell populations

Assessment of cells viability was performed either by counting of live and dead cells using the trypan blue (Lonza, Switzerland) exclusion method, or using the MTT [3-(4,5-dimethylthiazolyl-2-yl)-2,5-diphenyltetrazolium bromide] Cell Proliferation Assay (ATCC), according to manufacturer’s protocol.

Total RNA was extracted using RNeasy Mini kit (Qiagen) and reverse transcribed with NZY First-strand cDNA synthesis kit (NZYTech) according to manufacturer’s protocol. Quantitative PCR was performed using the following sets of primers: CIB2 forward: 5′-ACCAGGACTGCACCTTCTTC-3′; CIB2 reverse: 5′-TCTGGCATCTGGATGATGAG-3′; CIB1 forward: 5′-TTCCAGCACGTCATCTCCC-3′; CIB1 reverse: 5′-GCCACAGCTCAGCAGTAGAAA-3′; GAPDH forward: 5′-GGTGGTCTCCTCTGACTTCAACA-3′; GAPDH reverse: 5′-GTTGCTGTAGCCAAATTCGTTGT-3′. Reactions contained 1X SYBR Select Master Mix (Life Technologies), 300 nM each primer, and 150 ng of template DNA in a 25 μl volume. After initial incubations at 50 °C for 2 min and 95 °C for 2 min, 40 cycles of amplification (95 °C × 1 min; 60 °C × 1 min) were performed using an ABI 7500 Real-Time PCR System (Applied Biosystems). The standard curve method was applied for quantification of each amplicon. The levels of cDNA for CIB1 and CIB2 were normalized to that of GAPDH cDNA, and expressed as percentage of control (untransduced) cells.

### Production of HIV virus particles and challenge of shRNA cell populations

We used both replication competent and pseudotyped HIV particles to measure the susceptibility to HIV infection of shRNA-transduced cells populations. For replication competent HIV stocks, HEK 293 T cells were transfected with the plasmids pHIV-1_NL4-3_ or pHIV-1_NLAD8_ (AIDS reagents, contributors Dr. Malcolm Martin and Dr. Eric O. Freed) to produce X4-tropic and R5-tropic viruses, respectively. To produce pseudotyped viral particles competent only for a single cycle infection, the NL4-3Δenv^60^ or NL4-3ΔenvLuciferase^61^ plasmids were transfected in HEK 293 T cells together with a plasmid encoding either an X4 or an R5 HIV envelope glycoprotein complex^62^ (4:1 ratio), or with a plasmid encoding the VSV-G^59^ (9:1 ratio). Virus containing supernatants were collected 2 days post-transfection, clarified by centrifugation, aliquoted and stored at −80 °C. The p24 content was quantified by p24 ELISA (Innogenetics) prior to use.

To challenge shRNA-transduced Jurkat cells or primary CD4+ T-cells with replication competent HIV-1_NL4-3_ or HIV-1_NLAD8_, cells were resuspended in medium containing the indicated amounts of p24 and incubated for 2 h at 37 °C. During the 7 day infection assay in Jurkat cells, cells were diluted and medium was added at days 3 and 5. For Jurkat cultures, cells and supernatants were collected at days 3, 5 and 7 after infection for intracellular Gag (iGag) staining and p24 quantification, while for primary lymphocytes the cell and supernatant collections were performed on days 2, 3 and 4 after infection. A higher MOI was used to infect primary CD4+ T cells as compared to Jurkat cells.

To measure susceptibility to single-cycle infection, viruses pseudotyped with either VSV-G or HIV-1 envelope glycoprotein complexes were used to infect cells stably transduced with shRNAs. Pseudotyped NL4-3Δenv and NL4-3ΔenvLuciferase viruses were used to infect 1 × 10^5^ cells Jurkat cells or primary T-cells, respectively. Forty hours after infection, viral infectivity was measured either by intracellular Gag (iGag) staining as described below or by determining luciferase activity in target cells as described in^63^.

### Construction and expression of shRNA-resistant mRNA encoding CIB proteins

To verify that the effect on HIV infection was due to reduced expression of CIB1 and CIB2 mRNA, we conducted gain of function experiments in which expression of CIB1 or CIB2 was induced in shRNA transduced cells. To this end, we first constructed plasmids expressing CIB1 or CIB2 mRNA, and modified the plasmids to render the mRNA resistant to shRNA knockdown, and then co-transduced cells with lentiviral vectors expressing shRNA and vectors expressing the resistant form of CIB1 or CIB2 mRNA. To this end, total RNA was isolated from cultured Jurkat E6-1 cells using the RNeasy Total RNA Kit (Qiagen), and reverse transcribed with NZY First-strand cDNA synthesis kit (NZYTech) according to manufacturer’s protocol. CIB2 cDNA was amplified using the Phusion High-Fidelity DNA Polymerase (Life Technologies) and primers 5′-CTACTttcgaaATGGGGAACAAGCAGACCA (forward) and 5′-TGCATggatccTCAGATCCGGATGTGGAAA (reverse). The amplified CIB2 cDNA (0.5 kB) was digested with the Bsp119I and BamH1 restriction endonucleases and ligated into the pRRLSIN.cPPT.PGK-GFP.WPRE expression vector (Addgene) previously digested with the same endonucleases, producing pPGK-CIB2-eGFP. When transfected into human cells, this plasmid induces the expression of CIB2 cDNA under control of the phosphoglycerate kinase (PGK) promoter. To produce a vector allowing expression of a shRNA-resistant cDNA, a 0.6 kb CIB1 sequence was synthesized (GenScript) containing 6 silent mutations within the sequence targeted by CIB1 shRNAs and Bsp119I and BamHI restriction sites at the extremities. This CIB1 cDNA (see Supplementary Fig. S1) was cloned into pRRLSIN.cPPT.PGK-GFP.WPRE as described above, producing pPGK-CIB1-eGFP.

To produce lentiviral stocks that express cDNA for CIB1 or CIB2, we co-transfected HEK 293 T cells with pPGK-IRES-eGFP containing cDNA sequences, the R8.74 packaging vector and the VSV-G envelope vector.

For the phenotypic reversion experiments, 1 × 10^6^ Jurkat E6-1 cells were co-transduced with 50 ng of shRNA lentiviral vectors targeting CIB1 or CIB2 and 300 ng of the corresponding pPGK-CIB-eGFP lentiviral vector for 2 h at 37 °C and transduced cells were selected with puromycin as described above. Cells were then challenged by HIV, as described above.

### Viral release and infectivity assays

HeLa cells previously transduced with shRNAs were transfected with 1 μg of pHIV-1_NL4-3_ or pHIV-1_NL4-3_ΔVpu^64^, using jetPEI reagent. Supernatants and cell lysates were harvested 24 h post-transfection. Cellular debris was cleared by centrifugation and cells were lysed using medium with 10% NP40. p24 content was determined by ELISA (Innogenetics) and relative viral release was calculated by dividing the supernatant p24 by the total p24 (supernatant + cell lysate). To determine the infectivity of released virions, P4C5 cells were seeded in 96-well plates and 24 h later were infected with 1 and 2 ng of p24 of supernatants harvested from transfected HeLa populations. Forty hours post-infection, cells were lysed to evaluate β-galactosidase expression by a colorimetric assay based on the cleavage of chlorophenolred-β-D-galactopyranoside (CPRG; Roche)^65^. Preliminary experiments using a variety of different virus inputs demonstrated that using 1 and 2 ng of p24 would allow the detection of differences in infectivity of 25%. Three independent experiments, each performed in triplicate, were conducted.

### β-Lactamase-Vpr assay

The efficiency of viral entry into target cells was evaluated with the β-lactamase-Vpr assay, as previously described^35^. Virus stocks were produced by co-transfection of HEK 293 T cells with pHIV-1_NL4-3_Δenv, a plasmid coding for VSV-G or HIV-1 envelope glycoproteins and a plasmid encoding the Vpr gene fused to the β-lactamase gene (a kind gift from Michael D. Miller) in a 3:1:1 ratio, and the virus preparations were concentrated 10-fold by ultracentrifugation. As a control, virus stocks expressing a HIV-1 Env with the F522Y mutation, which prevents viral entry, were also prepared. Primary shRNA-transduced cells were exposed to the virus preparation for 4 h at 37 °C. Cells were then washed and loaded with the CCF2 substrate (CCF2-AM loading kit, Invitrogen) in the presence of 1.8 mM probenecid (Sigma-Aldrich). Cells were incubated overnight at 16 °C, washed with PBS containing 1% BSA and 0.05% Saponin (Sigma-Aldrich) and fixed with paraformaldehyde (PFA). The cleaved CCF2 fluorescence was measured by flow cytometry on a FacsCanto II system with FACSDiva software (BD Bioscience). FlowJo, version 10 (Tree Star), was used to analyze and quantify the data.

### Flow cytometry

For intracellular staining, cells transduced with vectors expressing shRNAs were fixed with 2% PFA, blocked and permeabilized with 1% BSA and 0.05% saponin (Sigma-Aldrich) in PBS for 10 min. Intracellular Gag (iGag) staining was performed with a phycoerythrin-conjugated mouse anti-Gag mAb (KC57-RD1; Beckman Coulter) in infected shRNA-transduced cells. The percentage of Gag positive cells was determined by flow cytometry using a FACS-Calibur instrument with CellQuest software (BD Bioscience). Intracellular staining for talin was performed using an anti-talin antibody (Sigma-Aldrich; Ref. T3287) followed by Alexa Fluor 488-conjugated secondary antibody (Molecular Probes). For surface staining, shRNA-transduced cells were washed with PBS containing 1% BSA and stained with the following antibodies: anti-human CD4-APC (eBioscience, Ref. 17-0047), anti-human CD184-PE (CXCR4, BD Pharmingen, Ref. 555974), anti-human CCR5-APC (R&D Systems, Ref.FAB1802-A), anti-human CD3-Alexa Fluor 488 (BD Pharmingen, Ref. 557694), anti-human CD11a (BD Pharmingen, Ref. 555382), and anti-human α4-β7 (Act-1, AIDS Research and Reference Reagent Program, Division of AIDS, NIAID, NIH) The staining with the two last antibodies was followed by incubation with an Alexa Fluor 488-conjugated secondary anti-mouse IgG antibody (Molecular Probes). Stained cells were fixed with 1% PFA and acquired using a FACS-Calibur instrument (BD Bioscience) or a FacsCanto II instrument (BD Bioscience). FlowJo was used to analyze and quantify the data.

### HIV binding assay

Primary CD4+ T-lymphocytes were incubated with 100 μl of virus supernatant diluted in serum-free RPMI to contain 20 ng or 100 ng of p24 for 2 h at 4 °C with periodic mild agitation. After incubation, cells were washed with ice-cold phosphate buffered saline and pelleted by centrifugation. Cells were transferred to a new plate and washed to prevent p24 carryover. Pelleted cells were lysed with 10% NP40, and the amount of virus bound to cells was measured by p24 ELISA (Innogenetics).

### Cell-to-cell HIV transfer assay

Cell-to-cell viral transfer was measured by a flow cytometry-based assay as previously described^66^. Briefly, non-transduced activated primary CD4+ T-lymphocytes were infected using 100 to 500 ng of HIV-1_NL43_ to be used as donor cells. After 48 h of infection, donor cells were washed to eliminate cell-free virions and labeled with carboxyfluorescein diacetate succinimidyl ester (CFSE) (2.5 μM; Molecular Probes) for 10 min at 37 °C. Untransduced cells and those transduced with the indicated shRNAs were co-cultured with the CFSE-labeled donor cells (1:1 ratio). At different time points (4 h and 24 h), cells were fixed in 2% PFA, and the percentage of intracellular Gag-positive cells was determined by flow cytometry as described above. In our assay, detection of intracellular Gag in target cells results from the fusion of the viral and cellular membranes^34^, while the signal associated with viral material trapped in endocytotic vesicles is negligible. To serve as controls, untransduced cells were also treated in parallel with either 6.25 µM nevirapine (NVP) or 2.5 μM T-20.

### Western blotting

Equal numbers of cells transduced with shRNAs targeting CIB mRNAs or the control sh-SCRAM were lysed with Laemmli lysis buffer (Sigma-Aldrich) containing protease inhibitors (Thermo Scientific). Proteins were electrophoresed into 12% polyacrylamide gels and transferred to nitrocellulose membranes (Amersham Biosciences). Membranes were blocked with 5% milk in Tris-buffered saline (TBS) containing 0.1% Tween20 for 1 h at room temperature. The primary antibodies, indicated in the figures, were incubated overnight at 4 °C in blocking buffer, washed, and probed with horseradish peroxidase (HRP)-conjugated secondary antibodies (Bio-Rad). Mouse anti-glyceraldehyde-3-phosphate dehydrogenase (GAPDH) (Santa Cruz Biotechnology) was used as a loading control.

### Statistical analysis

Statistical analyses were performed using GraphPad Prism 6 software. The tests used are indicated in the figure legends. Values were considered significantly different if p ≤ 0.05.

## Additional Information

**How to cite this article**: Godinho-Santos, A. *et al*. CIB1 and CIB2 are HIV-1 helper factors involved in viral entry. *Sci. Rep.* **6**, 30927; doi: 10.1038/srep30927 (2016).

## Supplementary Material

Supplementary Information
