# Supplementary material for: CIB1 and CIB2 are HIV-1 helper factors involved in viral entry
Source: Sci Rep. 2016 Aug 4;6:30927. doi: 10.1038/srep30927 (PMC4973253; doi:10.1038/srep30927)
Supplement: Supplementary Information [file srep30927-s1.pdf]

CIB1 and CIB2 are HIV-1 helper factors involved in viral entry

Ana Godinho-Santos[1,2]*, Allan J. Hance[2,3], João Gonçalves[1]* and Fabrizio Mammano[2,3]

1 Research Institute for Medicines (iMed.ULisboa), Faculty of Pharmacy, University of Lisbon, Lisbon, Portugal

2 INSERM, U941, Paris, F-75010, France

3 Univ Paris Diderot, Sorbonne Paris Cité, F-75475, Paris, France

* corresponding authors:

acsantos@ff.ul.pt

jgoncalv@ff.ul.pt

Gene	shRNA name	shRNA sequence
**CIB1**	sh-CIB1-A	CCGGGTCTGAGATGAAGCAGCTCATCTCGAGATGAGCTGCTTCATCTCAGACTTTTT
	sh-CIB1-B	CCGGCCAGACTTTGCCAGCTCCTTTCTCGAGAAAGGAGCTGGCAAAGTCTGGTTTTT
**CIB2**	sh-CIB2-A	CCGGGTCCTTTCCTACTCCAGAAATCTCGAGATTTCTGGAGTAGGAAAGGACTTTTT
	sh-CIB2-B	CCGGCGGGAGAATCCCTTCAAAGAACTCGAGTTCTTTGAAGGGATTCTCCCGTTTTT

Supplementary Table S1. shRNA sequences used for CIB1 and CIB2 genes.

Start codon

Atgggggctcgggcagtcgcctgtccaaggagctgctggccgagtacca
ggacttgacgttcctgacgaagcaggagatcctcctttctgtgtacgtgg
ttttagctcctcacctggttgacaatgagcagcaggcaaggagtgggaat
gaacacacagggagaccgatcgctgagaacaccgacagttcccctctctc
taccagagcccacaggcggttttgtgagctgcttccccaggagcagcgga
gcgtggagtcgtcacttcgggcacaagtgcccttcgagcagattctcagc
cttccagagctcaaggccaaccccttcaaggagcgaatctgcagggtctt
ctccacatccccagccaaagacagccttagctttgaggacttcctggatc
tcctcagtgtgttcagtgacacagccacgccagacatcaagtcccattat
gccttccgcatctttgactttgatgatgacggaaccttgaacagagaaga
cctgagccggctggtgaactgcctcacgggagagggcgaggacacacggc
ttagtgc**GTCCGAAATGAAACAATTGAT**cgacaacatcctggaggagtct
gacattgacagggatggaaccatcaacctctctgagttccagcacgtcat
ctcccgttctccagactttgccagctcctttaagattgtcctg**tga**

Stop codon

Supplementary Figure S1. CIB1 CDS sequence of transcript a used to express CIB1 in sh-RNA-transduced cells. This sequence contains 6 silent mutations (in blue) within the sequence targeted by sh-CIB1-A (in caps) in order to be RNAi-resistant.

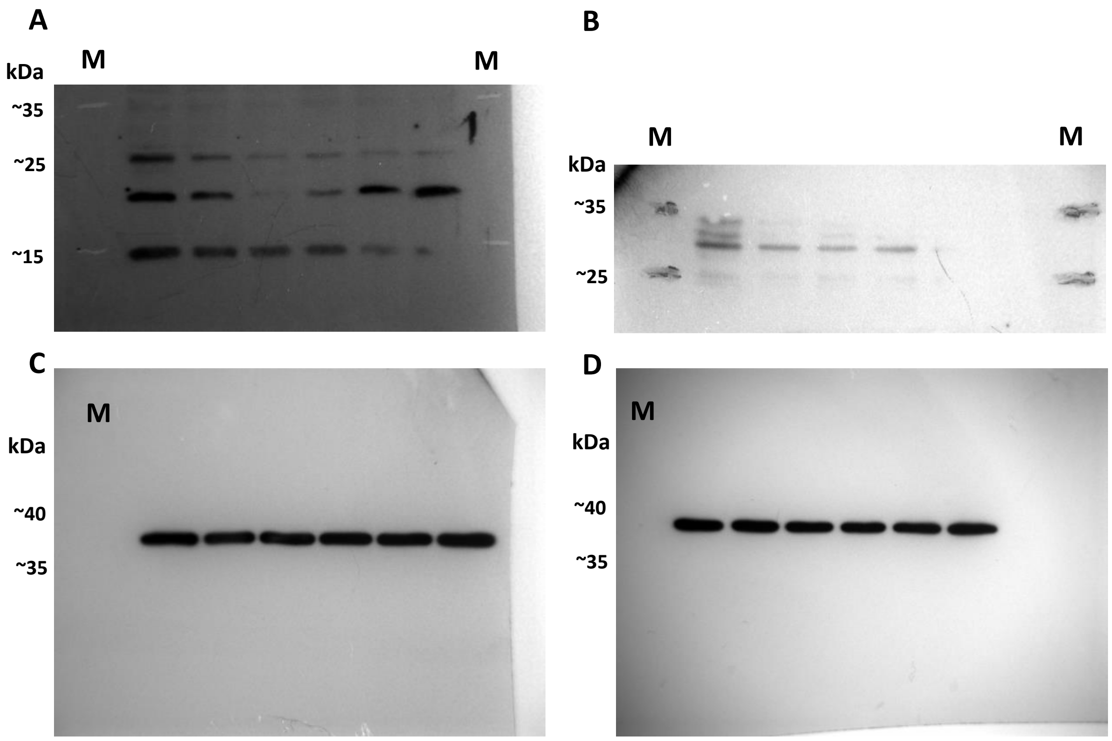

Supplementary Figure S2. Full-length blots from western-blotting to detect CIB1 protein (A) or CIB2 protein (B) using antibodies recognizing CIB1 (clone 791119, R&D systems) or CIB2 (clone CIB2C12B11, Abcam). Mouse anti-glyceraldehyde-3-phosphate dehydrogenase (GAPDH) (clone 6C5, Santa Cruz Biotechnology) was used as loading control in each run regarding detection of CIB1 (C) or CIB2 (D). The bands of the protein ladder are written in the side of each blot and the lane with the protein ladder is indicated with the letter M.
